# Carbon Nanomaterials: Application and Prospects of Urban and Industrial Wastewater Pollution Treatment Based on Abrasion and Corrosion Resistance

**DOI:** 10.3389/fchem.2020.600594

**Published:** 2020-11-12

**Authors:** XiaoQi Jia, Sheng Yuan, Bo Li, HongJiang Miu, Jing Yuan, CanFei Wang, ZuChao Zhu, YuLiang Zhang

**Affiliations:** ^1^Key Laboratory of Fluid Transmission Technology of Zhejiang Province, Zhejiang Sci-Tech University, Hangzhou, China; ^2^Key Laboratory of Air-Driven Equipment Technology of Zhejiang Province, Quzhou University, Quzhou, China; ^3^Hangzhou Weiguang Electronic Co., Ltd, Hangzhou, China; ^4^Hangzhou Dalu Industry Co., Ltd, Hangzhou, China; ^5^Zhejiang Institute of Mchanical & Electrical Engineering Co., Ltd, Hangzhou, China

**Keywords:** carbon materials, abrasion resistance, corrosion resistance, sewage pump, prospect

## Abstract

Environmental pollution as a result of urban and industrial wastewater has become an increasingly prominent issue. Rivers, lakes, and oceans that have become eutrophicated or polluted by suspended solids and hazardous substances in wastewater have endangered the environment. A root cause of this is the discharge of untreated urban and industrial wastewater into the ecosystem. As a solution to the pollution, wastewater treatment facilities have seen increasingly rapid development. Sewage pumps are designed to transport urban and industrial wastewater containing solid particles or hazardous substances to water treatment centers for purification and treatment. Sewage pumps are of great importance in the entire wastewater treatment system. Sewage in the environment where sewage pumps work usually contains sands, suspended particles, and plenty of saline ions. Flow passage components and sealing elements of the pump become vulnerable to abrasion and chemical corrosion, which further challenges operational stability of the pump. Research has remained focused on how to improve reliability of sewage pumps under severe conditions. Because of advances in materials science, the application of an increasing number of new materials has been witnessed, such as carbon-based composite materials and carbon nanomaterials, thanks to their fine self-lubrication performance, heat resistance, thermal and electrical conductivity, chemical stability, heat and seismic resistance, as well as plasticity. These properties contribute to enormous potential that new carbon-based composite materials and carbon nanomaterials have to offer in terms of corrosion resistance. This paper outlines application scenarios, research progress, and the prospect of new materials in sewage pumps.

## Introduction

As urban areas are becoming increasingly populated (McNabb, 2019) along with urbanization, it is expected that by 2050, a total of over 900 million people will be living in cities. By then, a soaring increase of domestic and industrial wastewater (Solon et al., 2017) will adversely cause wastewater pollution (Yan et al., 2015). Over 80% of wastewater in developing countries is discharged without treatment, resulting in river, lake, ocean, and soil eutrophication or pollution caused by suspended solids and hazardous substances. These sources of pollution are extremely detrimental to natural biology and mankind (Venkateswaran et al., 2007; Fan and Zhang, 2014; Savriama et al., 2015; Chen et al., 2017; Bougnom et al., 2019), contributing to untreated discharge of urban and industrial wastewater (Kadiya et al., 2013). In recent years we have witnessed increasingly-rapid development of wastewater treatment facilities as a solution to pollution issues (Sun et al., 2020, 2021). Among other solutions, sewage pumps, a type of centrifugal pump for sewage transport, are mainly designed to transport urban and industrial wastewater containing solid particles or hazardous substances, to water treatment centers for purification and treatment. The sewage pumps are of great importance in the entire wastewater treatment system. Conventional sewage pumps, in general, are made of stainless steel. Exposed to urban and industrial wastewater—which usually contains plenty of saline ions and slurry in solid particles—conventional materials, such as stainless steel, are vulnerable to abrasion and corrosion. Under the influence of solid particles and saline ions, flow passage components of the pump tend to fail partially, the size of the abrasion and corrosion increases and operational efficiency of the centrifugal pump decreases, resulting in enormous energy waste. Statistics show that electric energy consumed by the centrifugal pump accounts for 25–60% of total consumption (Sahoo and Guharoy, 2009). In the worst scenarios, the centrifugal pump may fail or trip, causing additional and significant maintenance costs (equals up to 50% of routine maintenance cost) (Sahoo and Guharoy, 2009). Abrasion and corrosion of the components from solid particles are deemed to be one of the leading contributors to pump failure (Kwok et al., 2000; Guo et al., 2005; Wood, 2006; Barik et al., 2009). Therefore, it is crucial to diagnose and take preventative actions at an early stage, to prolong service life of the pump and to reduce the economic loss to the greatest extent. Such abrasion may be defined as abrasion of structural members (mainly including blades of hydraulic turbine, pressure pipeline, hydraulic seal, and casing) from flowing solid particles that impact target surfaces at a very high speed (Padhy and Saini, 2009). As we are aware of the presence of abundant solids in pumped liquids, we should replace internal materials of the pump with materials with higher corrosion resistance, such as carbon steel, high-chromium iron, hardened stainless steel, ceramics, tungsten alloy, or other hard coatings that are frequently used as alternatives. Carbon-based composite materials and carbon nanomaterials, distinguished for fine mechanical property under high temperature, heat resistance, abrasion and corrosion resistance, and low thermal expansion (Schmidt et al., 1999; Wang et al., 2003; Chen et al., 2010; Sun et al., 2019), are good options for upgrading the reliability of sewage pumps. For this purpose, this paper elaborates on abrasion and corrosion resistance, chemical stability, and other aspects of carbon-based materials, and outlines their application prospects to improve reliability and service life of sewage pumps.

## Abrasion Resistance of Carbon-Based Composite Materials

Abrasion of metallic materials refers to the materials' loss of volume as a result of corrosion, cavitation, fouling, particle surface impact, etc. Centrifugal sewage and slurry pumps are often observed with material loss from particle impact, corrosion, erosion, etc. Depending on the shape, pump abrasion from particles contained in wastewater can be divided into sliding abrasion and impact abrasion (Wilson and Clift, 2006). Abrasion on such rotary parts as impellers is almost uniform, while that on a pump case is not (Roco et al., 1984), with abrasion at the cut-water particularly being the worst (Pagalthivarthi et al., 1990). Mueller et al. (1978) observed how badly a specimen stuck to the inner side of the pump case had been abraded, and they also assessed its corrosion. Among all the eight types of materials with different levels of hardness, they identified that the hard alloy stood out in the abrasion resistance test. Therefore, they proposed to coat the volute-shaped inner wall and impeller surface of the sewage pump with a layer of hard material, so that it could help the pump build higher resistance to abrasion at a low angle of impact. According to studies of Walker et al. (1994), impeller of the sewage pump was more likely to break down when exposed to larger solid particles, while the front shroud was more vulnerable to abrasion when exposed to smaller solid particles.

Owing to their superior abrasive resistance, new carbon-based materials demonstrate a great prospect of their application in sewage pumps for abrasion resistance purposes. Chen et al. (2019) designed an impeller of a centrifugal pump with new SiCNFs- carbon-carbon composite materials, and they found that abrasion resistance of the impeller became significantly upgraded, with abrasion loss considerably lower than that of ordinary carbon-carbon composite materials, particularly abrasion in a vertical direction. According to Shi et al. (2018) study on how the content of carbon fiber impacted anti-abrasion performance of a nickel-based composite coating, the structure of the material layers was found to be the most uniformly distributed and the abrasion resistance was 1.7 times that of the Ni-based coating without a carbon fiber, when the carbon fiber content reached 6%. Compared with an ordinary stellite without a carbon fiber, the type of stellite composite material prepared by Khoddamzadeh et al. (2014) with 5% carbon fiber, gained significant improvement in terms of hardness and abrasion resistance. In comparison with copper-based composite materials without a carbon fiber, as studied by Tang et al. (2008), the same materials with a short carbon fiber had a considerably higher abrasion resistance, and a dramatically lower friction coefficient and abrasion loss, yet with a slightly lower microhardness. Algul et al. (2015) finding reveals that the higher the content of graphene was, the significantly higher the microhardness and abrasion resistance of the nickel-based composite materials would be. Contributing to the higher abrasion resistance was the superior mechanical strength and lubricating effect that graphene had to offer. Wu et al. (2015) prepared a uniform graphene oxide coating on a low-carbon steel surface and discovered that graphene oxide in some concentrations were positive to abrasion resistance and microhardness of the coating. The graphite-copper composite material prepared by Moustafa et al. (2002) under a powder metallurgy process, demonstrated a lower abrasion rate and friction coefficient than pure copper and could withstand a 500N load when graphite content reached 20%. Du et al. (2004) abrasion test of Al_2_O_3_ composite materials with different contents of short fiber showed that, the lowest abrasion rate of the materials could be reached when the content of short fiber was 12%. Bushueva et al. (2019) studied texture, mechanical and tribological property of modified coating on chromium-nickel austenitic steel surface, and concluded that carbide could notably contribute to higher microhardness of materials and a 1.4 times higher abrasion resistance, when compared with ordinary materials without the coating. As for stainless steel, the surface of which was coated with reinforced nickel-based composite materials by laser cladding technique, Lei et al. (2017) studied and concluded that carbon fiber could work to effectively improve corrosion and abrasion resistance of a nickel-based coating. Suresh et al. (in press) managed to combine carbon fiber and aluminum oxide packing, and concluded that coating prepared by a thermal evaporation technique is an incredibly efficient solution to abrasion resistance under any load. From the study on abrasive performance of reinforced vinyl ester composite material with carbon fiber, Suresha and Kumar (2009) inferred that the abrasion rate of the material reached only 40% of the same material with glass.

## Corrosion Resistance of Carbon-Based Composite Materials

Centrifugal sewage pumps in urban, industrial, mining, and other applications are usually made of stainless steel 304 which primarily consists of iron, carbon, and at least 11% chromium. Among others, chromium is known as a corrosion-resistant element and helps improve anti-strip performance, strength, and abrasion resistance of materials. High corrosion resistance of alloy is dependent on a layer of invisible film, a passivation coating that is stable, durable, of adhesive power, and self-repair ability (Ilevbare and Burstein, 2003). Despite its very high corrosion resistance in a variety of mediums, austenitic stainless steel still could not avoid corrosive pitting when exposed to chlorides (Fossati et al., 2006). Chloridion is the main contributor to this corrosion (Meguid et al., 2000; Yang and Luo, 2001; El-Egamy and Badaway, 2004). Some theoretical explanations for the occurrence of this corrosive pitting, for example local acidification (Galvele, 1978), depassivation-repassivation behaviors (Richardson and Wood, 1970; Dawson and Ferreira, 1986), point defect model (Chao et al., 1981; Macdonald and Urquidi-Macdonald, 1985), and anion-permeable membrane/migration (Okamoto, 1973), are available, but they all suppose that chloridion is absorbed onto the metal surface. Coating works to isolate the steel surface from corrosive environment and prevents diffusion of oxygen, vapor or ion (Liu et al., 2003). As an inorganic non-metallic material, carbon fiber remained unaffected by oxygen in the wastewater and avoided falling off under the effect of ions in the solution on one hand; and served as an anti-corrosion film and provided fine abrasion resistance and hardness on the other hand, making metal protection less vulnerable to degradation from abrasion and falling of solid particles.

Ge et al. (2020) added carbon fiber packing into zinc-rich epoxy coating to increase its electrical conductivity, and found that this coating could protect copper-based material in 3.5% NaCl solution for up to 50 days, much longer than the protection duration by carbon-fiber-free zinc-rich epoxy coating. Liu et al. (2016) introduction of 0.5% graphene to the epoxy coating promoted its resistance to corrosion, with the corrosion rate being an order of a magnitude lower than graphene-free coating. According to Zhou et al. (2019) findings, graphene oxide worked to effectively prolong the duration of zinc-rich epoxy cathodic protection and fully protected metals, thanks to its superior conductivity. After adding a 0.5% graphene nanosheet, Chen et al. (2017) made a conclusion that the excellent mechanical and barrier properties of graphene nanosheet could considerably improve corrosion resistance of coating. Lei et al. (2017) studied and proved that carbon fiber on the surface could work to upgrade nickel-based coating's resistance to corrosion in an effective way (e.g., corrosion current density of composite coating was only 7% that of nickel-based coating). Cheng et al. (2019) proposed a type of new technology for preparing composite films with adhesive protein and graphene, as their graphene film demonstrated strong adhesive force to carbon steel, which could notably improve corrosion resistance of carbon steel. Jiang et al. (2017) adopted a ultrasonic vibration direct-current electrodeposition technique and prepared a nanometer Ni-Mo/graphene oxide composite material on the surface of low-carbon steel, and proved that the material had been considerably improved in terms of resistance to corrosion and oxidization.

## Conclusions and Prospect

In light of their extensive application in wastewater treatment systems and the extremely adverse environment that they are intended to work in, it has become a an area of focus for researchers to try figure out how to improve the sewage pumps' resistance to abrasion and corrosion, reliability, and service life. In pursuit of solutions, researchers have been devoted to studies on abrasion and corrosion resistance of carbon fiber, carbon-carbon composite materials, and carbon nanocomposite, as well as on how to reduce abrasion and corrosion from particles. Unlike conventional metal materials, weaknesses, however, persist and limit continuous high-quality production of carbon composite materials, as their development and application started at a later stage. Considering that a great many novel carbon composite materials are still under study and undergoing experiments, and the growth mechanism of some carbon materials is yet to be elucidated, carbon materials are restricted in terms of mass production and application without having a clear understanding of how to restructure or control the material structure. Therefore, it becomes imperative to delve into abrasion and corrosion-resistant carbon materials and to develop distinctive carbon materials on the basis of their features and superior performance. Future studies should focus on how to develop special carbon materials with more resistance to heat, abrasion and corrosion, chemical stability, high weight-rigidity ratio, fine self-lubrication, etc. In the future, the application of carbon nanocomposites in ultra-high abrasion-resistant coatings, stable and durable seals, and bearings made of special materials in the field of centrifugal pump abrasion from solid particles will become the hotspot.

## Author Contributions

XJ and ZZ contributed to the conception of the study. XJ and SY wrote the article. BL, HM, JY, and CW researched the information. SY and YZ edited the article. All authors contributed to the article and approved the submitted version.

## Conflict of Interest

BL was employed by the company Hangzhou Weiguang Electronic Co., Ltd, Hangzhou, China. HM was employed by the company Hangzhou Dalu Industry Co., Ltd, Hangzhou, China. CW was employed by the company Zhejiang Institute of Mchanical & Electrical Engineering Co., Ltd, Hangzhou, China. The remaining authors declare that the research was conducted in the absence of any commercial or financial relationships that could be construed as a potential conflict of interest.
